# Antioxidant Power on Dermal Cells by Textiles Dyed with an Onion (*Allium cepa* L.) Skin Extract

**DOI:** 10.3390/antiox10111655

**Published:** 2021-10-21

**Authors:** Claudia Volpi, Desirée Bartolini, Virginia Brighenti, Francesco Galli, Matteo Tiecco, Federica Pellati, Catia Clementi, Roccaldo Sardella

**Affiliations:** 1 Department of Medicine and Surgery, University of Perugia, Piazzale Severi, 06132 Perugia, Italy; claudia.volpi@unipg.it; 2 Department of Pharmaceutical Sciences, University of Perugia, Via Fabretti 48, 06123 Perugia, Italy; desirex85@hotmail.it (D.B.); francesco.galli@unipg.it (F.G.); roccaldo.sardella@unipg.it (R.S.); 3 Department of Life Sciences, University of Modena and Reggio Emilia, Via G. Campi 103, 41125 Modena, Italy; virginia.brighenti@unimore.it; 4 Department of Chemistry Biology and Biotechnology, University of Perugia, Via Elce di Sotto 8, 06123 Perugia, Italy; matteotiecco@gmail.com; 5 Center for Perinatal and Reproductive Medicine, University of Perugia, Santa Maria della Misericordia University Hospital, 06132 Perugia, Italy

**Keywords:** *Allium cepa* L., onion, polyphenols, biofunctional textiles, mordant, ROS, antioxidant, skin

## Abstract

In this study, the phenol loading and antioxidant activity of wool yarn prepared with the aqueous extract of onion (*Allium cepa* L.) skin was enhanced by implementing the dyeing process with the use of alum as a mordant. Spectrophotometric and chromatographic methods were applied for the characterization of polyphenolic substances loaded on the wool yarn. The antioxidant/anti-inflammatory properties were evaluated by determining the level of intra- and extra-cellular reactive oxygen species (ROS) production in keratinocytes and dermal fibroblasts pre-treated with lipopolysaccharide put in contact with artificial sweat. An elevated dye uptake on wool was observed for the pre-mordanted sample, as demonstrated by high absorbance values in the UV-Visible spectral range. Chromatographic results showed that protocatechuic acid and its glucoside were the main phenolic acid released in artificial sweat by the wool yarns, while quercetin-4′-glucoside and its aglycone quercetin were more retained. The extract released from the textile immersed in artificial sweat showed a significant reducing effect on the intra-and extracellular ROS levels in the two cell lines considered. Cytofluorimetric analyses demonstrated that the selected mordant was safe at the concentration used in the dyeing procedure. Therefore, alum pre-mordanted textiles dyed with onion-skin extracts may represent an interesting tool against skin diseases.

## 1. Introduction

In recent years, environmental sustainability has become one of the key features of the broader concept of sustainable development [1,2]. In particular, a great effort is spent to reduce waste deriving from agricultural and food processing procedures in a circular economy perspective, meaning the recycle and reuse of waste material as the feedstock for production of new ones [1]. Agricultural waste, intended as the not-edible parts derived from food processing, can indeed represent a source of high-value-added products that can be exploited in several fields [1,2].

In this perspective, *Allium cepa* L. (onion) processing waste has great importance, as it has recently become the second most abundant horticultural crop in the world [3,4,5,6]. Special attention is paid to onion skin waste, being the major one in onion processing not yet been valorized as a by-product despite its rich composition in polyphenolic compounds, encompassing quercetin derivatives and phenolic acids [3,4,5,6].

In this scenario, extracts derived from onion skin represent a rich source of colored bioactive compounds to be used for the sustainable production of colored textiles also endowed with specific health-promoting properties on human skin [5,7]. These smart materials are usually referred to either as biofunctional textiles or cosmeto-textiles [8,9,10,11]. Taking advantage of the slow and continuous release of bioactive molecules to human skin through the direct contact with the epidermal tissue [12,13,14], these kinds of materials are increasingly gaining the interest of the scientific community as an innovative vehicle for the local treatment of skin diseases. Besides their traditional use as coloring agents, natural dyes are gaining popularity for the numerous functional properties which are capable of imparting a fibrous substrate. Moreover, these colored phytocomplexes are particularly suitable for the production of biofunctional textiles because of their low toxicity, high biocompatibility, and biodegradability combined with their minimum environmental impact [5,8,10].

Being that onion skin is rich in flavonoids and phenolic acids, its extract is characterized by an intrinsic strong antioxidant activity through a radical scavenging mechanism [15,16,17,18] that has been demonstrated to protect human skin from lipoperoxidation caused by UV radiation [5], while it may also help both to slow down aging-related processes and to inhibit the onset of degenerative diseases [19]. These beneficial biological activities are entirely preserved when the bioactive molecules are transferred from the extract to the textile substrate [20]. 

In view of possible future usage of textiles biofunctionalized with dried onion skin extracts in medical practice, it is essential to have a deep knowledge of this material both from a chemical and biological point of view. In this framework, it also covers crucial importance to progressively ameliorate the loading ability of textile fibers along with their release behavior in order to ensure the effectiveness, safety, and reproducibility of the beneficial properties. Therefore, efforts are being made for the continuous technological improvement of the textile production process. Thus, for instance, to enhance the loadability of biofunctional textiles, several strategies are being comparatively evaluated, spanning from relying upon microencapsulation or modification of the textile surface to the use of additives aimed at enhancing the interaction between the bioactive molecule and the textile [5,8,21,22,23,24]. Accordingly, a previous study carried out in our laboratories underlined how the addition of a zwitterionic surfactant to the dyeing bath can positively improve the loadability of wool yarn by the phenolic-based phytocomplex extracted from the dry skin of the *Dorata di Parma* onion cultivar [8]. In the same work, we also demonstrated that the zwitterionic additive did not impair the effective transfer of the biomolecules pool from the textile to the skin [8]. The assessment of their related biological properties comes accordingly with the definition of the loading and release behavior of the textile. 

In the light of all the above, in the present study, dry onion skin waste from the *Dorata di Parma* cultivar was selected as the source of biomolecules for the production of colored and biofunctional wool yarns with antioxidant properties. In line with our previous studies [5,8], dyeing experiments were performed according to historical and environmentally friendly procedures by dipping and boiling wool yarn in the aqueous extract obtained from onion skin. In order to increase the loading capacity of active principles on the fiber substrate and thus to attempt the enhancement of the functional behavior, dyeing was performed here using potassium aluminum sulfate (alum)—KAl(SO_4_)_2_ × 12H_2_O—as a mordant with three different application methods, namely pre-, meta- and post-mordanting. In the former, the textile was pretreated with a mordant solution before the dyeing process, whereas in the others the mordant was directly added to the dye bath during (meta-) and after (post-) the dyeing step. Therefore, with the two last methods, the mordant and dye application take place in the same bath with a consequent saving of energy and water consumption. Unlike not-mordanted dyed wool (T1) and micelle-dyed sample (T2), where weak interactions are expected to be established between the dyeing molecules and the textile substrate, in the presence of alum mordant, the aluminum ions form strong covalent and coordination bonds mainly involving the hydroxyl and carbonyl groups of dyes and woolen yarn amine functionality, thus acting as a solid chemical bridge between the two systems.

The optical properties, the chemical composition, and the biofunctional behavior of the colored aqueous extract and textiles were evaluated through a multi-analytical approach based on the combined use of chromatographic techniques and a series of UV/Vis spectroscopic methodologies. 

The method optimized to produce a new textile endowed with enhanced biofunctional properties than that previously described [5] is herein described. The valuable antioxidant capacity of the best textile was demonstrated through its capability to reduce significantly the production of extracellular and intracellular reactive oxygen species (ROS) in human keratinocytic and dermal human fibroblast cell lines pre-treated with lipopolysaccharide (LPS). Cytotoxicity and pro-apoptotic activity of alum aqueous solutions, at the concentration used in the dyeing procedure, were also evaluated to gain a deeper insight into the safety in use of the bio-functional textiles.

The present study may pave the way toward the development of new-generation textiles with skin-health-promoting properties by following sustainable production in a circular economy perspective.

## 2. Materials and Methods

### 2.1. Chemicals and Reagents

Dry onion skins from the “Dorata di Parma” cultivar were kindly provided by Cannara Onion Producers Union (Consorzio dei Produttori della Cipolla di Cannara, Italy) and processed without further pre-treatments. The raw wool yarn used in this study was provided by the cultural association “Franco Brunello” (Enego, Italy). Brij^®^S10 non-ionic surfactant for the scouring of wool yarn was purchased from Sigma-Aldrich (Milano, Italy). 3-(N,N-dimethyltetradecylammonio)propane-1-sulfonate (SB3-14) were purchased from Fluka (Milan, Italy) and purified twice by crystallization from a methanol/acetone mixture. Alum, that is, aluminum potassium sulphate dodecahydrate (AlK(SO_4_)_2_ × 12H_2_O, 99.5%) was purchased from Sigma-Aldrich. The Folin-Ciocalteu reagent, sodium chloride (NaCl), gallic acid (GA), lactic acid, urea, ammonia solution 32%, sodium carbonate (Na_2_CO_3_), and ethanol (EtOH) were purchased from Merck Life Science (Merck KGaA, Darmstadt, Germany). Water (H_2_O) was purified by using a Milli-Q Plus185 system from Millipore (Milford, MA, USA). DCFH-DA probe was purchased from Sigma-Aldrich and Amplex™ Red Hydrogen Peroxide/Peroxidase Assay Kit from Invitrogen. 3-(4,5-dimethylthiazol-2-yl)-2,5-diphenyltetrazolium bromide, MTT, was purchased from Sigma Aldrich.

The investigated textiles were the following: T1, dyed wool yarn without any additive; T2, wool yarn dyed in the presence of the SB3-14 zwitterionic surfactant; T3, wool yarn pre-mordanted with potassium aluminum sulfate; T4, wool yarn post-mordanted with potassium aluminum sulfate; T5, wool yarn meta-mordanted with potassium aluminum sulfate; and T6, wool yarn pre-mordanted with potassium aluminum sulfate and dyed in the presence of SB3-14 zwitterionic surfactant.

### 2.2. Scouring and Dyeing Procedure

The wool yarn was washed with a Brij^®^S10 non-ionic detergent solution (5 g/L, yarn to liquid ratio 1:100 *w*/*v*), according to a previously published method [5]. This sample is referred to as untreated wool (UW) throughout the text.

The preparation of the extract for wool-dyeing experiments was performed following a previously reported procedure [5]. Briefly, for a total of 16 g of wool to be dyed, 1600 mL H_2_O were added to 16 g of onion skin (100% onion skin with respect to the dry wool weight) and the mixture was kept under magnetic stirring at 95 °C for 1 h. The resulting extract was filtered through paper filter, then divided into six fractions, each of them being readily used as a dye bath to separately dye 1 g of scoured (T1, T2, see below for details) or mordanted (T3–T6, see below for details) wool yarn. In this way, the six different dyeing procedures leading to samples T1–T6 were performed with the same extract, starting with an identical chemical composition. For each dyeing experiment, 1 g of wool was dipped into 80 mL of onion skin aqueous extract (1:80 *w*/*v* liquid ratio) previously heated at 40 °C. The temperature was then increased to 95 °C and kept constant for 60 min. The wool was then removed, rinsed several times with H_2_O until the aqueous phase appeared uncolored, and air dried in the dark at room temperature. Sample T1 was produced following this procedure for 1 g of scoured wool, whereas samples T2 and T6 were prepared by adding SB3-14 surfactant to the dye bath until a concentration of 2.5 × 10^−3^ M was reached. Samples T3–T6 were treated with alum (0.25 g of alum for 1 g of dry yarn weight), following three different mordanting procedures. Samples T3 and T6 were previously pre-mordanted before the dyeing step [25]. In particular, for 1 g of wool, 0.25 g of alum was dissolved in 50 mL H_2_O and heated at 40 °C; then, the scoured wool yarn was added, and the temperature increased to 95 °C. The bath was kept constant at this temperature for 60 min under slight stirring in order to promote a homogeneous mordanting; then, it was allowed to cool to room temperature. The treated wool was then rinsed several times with H_2_O, allowed to dry in the dark at room temperature, and subsequently subjected to the dyeing process. Sample T4 was post-mordanted by removing the wool yarn from the dye bath after 30 min of dyeing and dissolving the mordant in the dye bath under stirring. The wool was then dipped again into the dye bath and allowed to dye for a further 30 min. Sample T5 was simultaneously (meta-) mordanted by dissolving the mordant directly in the dye bath before the wool yarn to be dyed was added.

For each sample, the obtained dyed wool yarn was carefully rolled around a Plexiglas sheet to have a 3 × 1 cm skein with a flat and homogeneous surface, which was therefore adequate for in situ reflectance and colorimetric measurements.

### 2.3. Photophysical Measurements

Reflectance spectra on solid samples were performed using a portable instrument composed of Avantes parts and equipped with a quartz fiber optic system already described in a previous paper [26]. The reflectance spectra were expressed in terms of pseudoabsorbance, A′(λ), according to Equation (1) [27]:

A′(λ) = log[1/(0.01∙R(λ))](1)

where, R is the measured reflectance at each specific wavelength λ. Colorimetric measurements on dyed samples (lightness (L*), redness–greenness value (a*), and yellowness–blueness value (b*)) were carried out by the light-reflectance technique on a Konica Minolta CM-700d spectrophotometer (Tokyo, Japan) under D65 illuminant and 10° standard observer. Four measurements were made for each sample recording the percentage reflectance values over the 350–750 nm spectral range and data reported are expressed as mean ± SD of four independent experiments.

### 2.4. Preparation of Artificial Sweat

The artificial sweat was prepared according to the reference test method EN 1811:2011. The aqueous solution contained 0.5% (*w*/*v*) sodium chloride, 0.1% (*w*/*v*) lactic acid, and 0.1% (*w*/*v*) urea. The pH was adjusted to 6.5 with ammonia solution.

The following procedure was applied in analogy to a previous study [8]. About 3 mg of dyed textile were put into a 0.2 mL of artificial sweat and kept at 37 °C for 2 h. Then, the textile was removed from the solution and allowed to dry at room temperature. The same textile was re-immersed in another aliquot of 0.2 mL of artificial sweat and kept at 37 °C for 2 h. The same procedure was repeated for five cycles, for a total of six determinations. The total phenol content TPC was determined with the Folin-Ciocalteu assay for each of the seven solutions. All the UV spectra were recorded at 25 °C with a Varian Cary 100 (Varian Inc., Palo Alto, CA, USA) dual-beam, dual-chopper spectrophotometer.

### 2.5. Determination of Total Phenol Content (TPC) by the Folin-Ciocalteu Method

The Folin-Ciocalteu reagent was diluted 10-fold with H_2_O. A definite volume of extract (0.1 mL) was mixed with 0.75 mL of the diluted Folin-Ciocalteu reagent and incubated in the dark for 10 min at room temperature. Then, 0.75 mL of 2% Na_2_CO_3_ (*w*/*v*) aqueous solution were added. The mixture was kept in the dark for 3 h before measuring the absorbance at 765 nm. The content of total phenolics was determined by using an analytical curve prepared with gallic acid (GA) solutions previously treated in the same way as for the real samples. Therefore, results were expressed as mg of GA equivalents/g textile. Data reported are expressed as mean ± SD of three independent experiments.

### 2.6. Quantification of the Main Polyphenols by Means of HPLC-UV

HPLC analyses were performed on an Agilent Technologies (Waldbronn, Germany) modular model 1260 Infinity II system consisting of a vacuum degasser, a quaternary pump, an autosampler, and a UV/Vis detector. The chromatograms were recorded using an Agilent OpenLab CDS ChemStation Edition (Rev. C.01.10). The analysis of phenolic acids and flavonols was carried out on an Ascentis Express C_18_ column (150 × 3.0 mm I.D., 2.7 µm, Supelco, Bellefonte, PA, USA). The mobile phase was composed of 0.1% HCOOH (*v*/*v*) in both (A) water and (B) ACN. The gradient elution was modified as follows: 0–5 min 3% B, 5–45 min from 3% to 50% B. The post-running time was 10 min. The flow rate was 0.4 mL/min. The sample injection volume was 20 µL. UV chromatograms were acquired at 254 nm. Three injections were performed for each sample [5,8].

### 2.7. Cell Lines and Culture Conditions

Human skin keratinocytes (NCTC 2544, Zooprophylactic Institute of Brescia, Italy) and human fibroblasts (HuDe) were grown in minimum essential medium (D-MEM, Gibco, Invitrogen) enriched with glutamax (Lonza), streptomycin (100 µg/mL)-penicillin (100 µL/mL) solution (1% *v*/*v*), and FBS (10% *v*/*v*; Gibco, Invitrogen) at 37 °C and 5% CO_2_. Primary Human dermal fibroblasts, HDF (106-05A, Sigma-Aldrich) from adult skin were grown in Fibroblast Growth Medium (116-500, Sigma-Adrich) containing 10% FBS at 37 °C and 5% CO_2_.

For cell maintenance, the culture medium was replaced with a fresh medium every 48 h, until ~80% cellular confluence was achieved. The cells were washed with phosphate buffer solution (PBS, Euroclone) and were treated with trypsin/EDTA (1×, Euroclone) solution to induce their detachment. The detached cells were collected in a centrifuge tube and centrifuged (1500 rpm) at room temperature for 5 min with an Eppendorf Centrifuge 5810. Finally, before in vitro experiments, a fresh D-MEM medium was used to re-suspend the pellet and then skin cells were plated 10,000 cells/well into 96-wells for the experiments reported below.

The murine monocyte/macrophage cell line RAW 264.7, obtained from the American Type Culture Collection (ATCC, Manassas, VA, USA), were cultured according to standard procedures in Roswell Park Memorial Institute 1640 medium (RPMI-1640), supplemented with 10% heat inactivated Fetal Bovine Serum (FBS), 2 mM of L-glutamine, and antibiotics (100 U/mL penicillin, 100 μg/mL streptomycin; Gibco, Invitrogen, Carlsbad, CA, USA). Cells were cultured at 37.0 °C in 5% CO_2_.

### 2.8. Analysis of Cellular Viability and Apoptosis of Alum on RAW 264.7 Cells

The murine monocyte/macrophage cell line RAW 264.7 was used to investigate alum cytotoxic and pro-apoptotic activities. RAW 264.7 cells were cultured at 37.0 °C in 5% CO_2_ for 24 h, in the presence (medium alone) or absence of alum. Cells were re-suspended at the concentration of 1.5 × 10^6^ cells/mL in a 12-well plate. The percentage of live, apoptotic, and dead cells was determined by using Annexin V Apoptosis Detection Kit PerCP-eFluor™ 710 and FVD (Fixable Viability Dye eFluor™ 780; eBioscience, San Diego, CA, USA), according to the manufacturer’s instructions. Specifically, each cell sample was washed and suspended in 100 μL of phosphate-buffered saline before the staining. A dilution 1:1000 of FVD was then added to the sample and incubated at 4 °C for 30 min in the dark. Annexin V-PerCP, diluted 1:20, was added and incubated for 15 min at RT in the dark. Flow cytometry analysis was performed within 4 h. Data are representative of two different experiments.

### 2.9. Cell Viability of Keratinocytes and Dermal Cells

Cell viability was evaluated on NCTC 2544, HuDe and HDF cells using the MTT test. Cells (0.1 × 10^5^ cells/well) seeded in 96-well plates were pre-incubated in medium with LPS (50 ug/mL; Sigma-Aldrich) for 6 h and then incubated with tissue T3 in sweat or water for 24 h. Cell viability was then assessed by 3-(4,5-dimethylthiazol-2-yl)-2,5-diphenyltetrazolium bromide (MTT, Sigma Aldrich) test accordingly with Mosmann T, 1983 [28]. Data reported were mean ± SD of three independent experiments run in triplicate.

### 2.10. Intracellular ROS Production (DCFH-DA Assay)

Cells (0.1 × 10^5^ cells/well), seeded in 96-well black plates, were pre-incubated in medium with LPS (50 µg/mL; Sigma-Adrich) for 6 h and then incubated with tissue T3 in sweat or water for 24 h. Intracellular ROS were assessed using the fluorescent probe 2′,7′-dichlorofluorescein diacetate (DCFH-DA; Sigma-Adrich) according to [29]. Data were mean ± SD of three independent experiments run in triplicate.

### 2.11. Extracellular ROS Production (Amplex Red Assay)

Cells (0.1 × 10^5^ cells/well), seeded in 96-well black plates, were pre-incubated in medium with LPS (50 µg/mL; Sigma-Adrich) for 6 h and then incubated with tissue in sweat or water for 24 h. Extracellular ROS were determined as H_2_O_2_ with a microplate assay procedure utilizing the Amplex™ Red Hydrogen Peroxide/Peroxidase Assay Kit (Invitrogen). Briefly, 100 μL cell supernatant was placed in 96-well plates and incubated in a humidified atmosphere with 5% CO_2_ at 37 °C, with 25 μL of HRP (1 U/mL) and 25 μL of Amplex red for 10 min. Then, the fluorescence was measured at λ_excitation_ = 560 ± 20 nm and λ_emission_ = 585 ± 20 nm using a DTX880 Multimode Detector microplate reader (Beckman Coulter). The assay was calibrated with authentic H_2_O_2_ and two quality control samples (QCs). Data were mean ± SD of three independent experiments run in triplicate.

### 2.12. Statistical Analysis

Data were analyzed using a student’s *t*-test or one-way ANOVA (with multiple comparisons) using Graphpad Prism 9.0 software. A *p*-value < 0.05 was considered significant. Each experiment was performed at least three times. * *p* < 0.05; ** *p* < 0.01; *** *p* < 0.001.

## 3. Results and Discussion

### 3.1. UV/Vis Spectral Properties of Dyed Textiles

**Figure 1:**
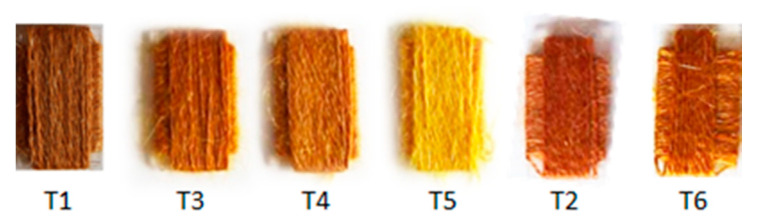
Dyed textile samples.

Textile samples submitted to different dyeing procedures showed a different color, as it is possible to notice from Figure 1.

**Figure 2:**
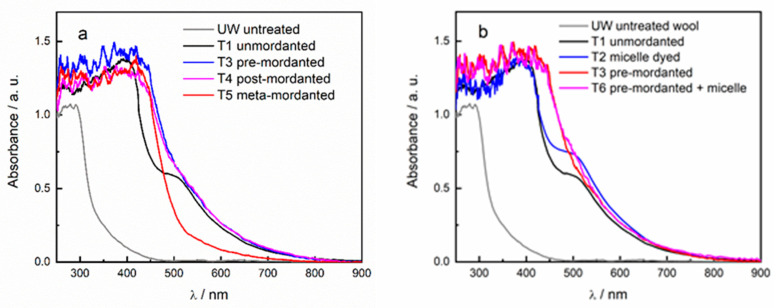
Absorption spectra of mordanted-dyed samples (**a**) and micelle-dyed samples (**b**) compared with that of the unmordanted-dyed (T1) sample and untreated wool (UN).

The absorption spectra of textile sample T1 (Figure 2a), dyed with the onion skin extract, show the characteristic flavonoid absorption band I, centered at 390 nm, and an unidentified bathochromic feature at 510 nm, probably due to flavonoid aggregates [5], resulting in a deep red-brown color as shown in Table 1 and described in a previous paper [5].

It was previously proven that the introduction of SB3-14 surfactant micelles in the dyeing step (sample T2) leads to a greater dye uptake, as demonstrated by higher absorbance values in the visible spectral range (Figure 2b) [8]. A different absorption profile was instead observed in all samples treated with alum mordant, where the flavonoid band I results shifted to longer wavelengths (about 10 nm) and centered at 405 nm, whereas the band at about 510 nm was not clearly distinguishable (Figure 2a). Samples T3 and T4, indeed, showed a broad tail that extended beyond 500 nm, and it is also responsible for the red-brown color (Figure 2 and Table 1). The absence of this absorption tail in the spectrum of sample T5 is responsible for its dark yellow color (Figure 1 and Figure 2a), as demonstrated by the higher value of the b* coordinate, thus indicating a lesser dye uptake with respect to the other mordanted samples. It is worth to notice that sample T3 showed higher absorbance values in the range 250–450 nm, thus revealing a greater dye content on wool fibers. The integration of pre-mordanting treatment with micelle dyeing did not lead to relevant improvements in terms of dye uptake, as shown by the very similar values of trichromatic coordinates and spectral features for samples T3 and T6.

### 3.2. Release Behavior of the Phenolic Compound from the Dyed Textile in Artificial Sweat

#### 3.2.1. Determination of the Total Phenol Content

Data shown in Table 2 indicate that an improved release of phenolic compounds is promoted by the zwitterionic surfactant, as already described in our previous publication (sample T1 vs. sample T2) [8]. The textile treated with the surfactant and pre-mordanted with potassium aluminum sulfate (sample T6) only apparently delivered less efficiently than sample T2 (Table 2). Indeed, the rather elevated standard deviation for the former sample (T6) makes the release behavior of this textile nearly overlapped to that of sample T2. This evidence clearly suggests an inhomogeneous impregnation of sample T2 with the phenolic extract.

As far as the three applied mordanting methods are concerned, data in Table 2 (T3–T5, respectively for the pre-, post-, and meta-mordanting treatment) readily highlight the pre-mordanting procedure as the one providing the best phenol release (T3). The release behavior by sample T5 is in good agreement with the UV/Vis spectral properties described in Section 3.1. Thus, although the meta-mordanting treatment is the most environmentally clean option among all those tested, it is less appealing for the production of biofunctional textiles.

**Figure 3:**
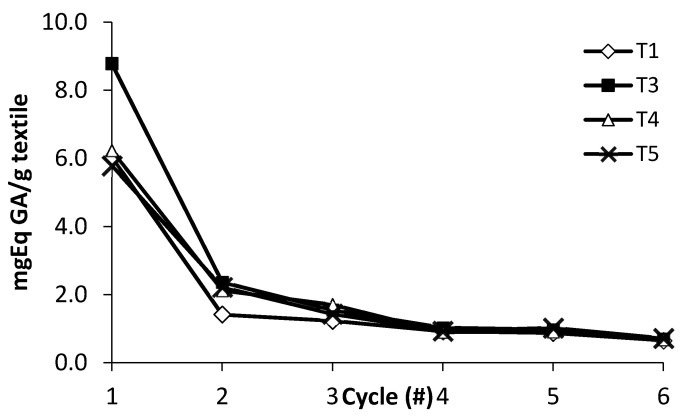
Release of the phenolics from textiles T1, T3, T4, and T5 in an artificial sweat solution.

Following a method already applied in a previous study [8], we virtually evaluated the release of phenolics from a selected number of the treated textiles, namely samples T1, T3, T4, and T5. All these samples share a common feature being untreated with the zwitterionic surfactant. Indeed, the use of this additive was found to be not necessary to improve the uptake of the biofunctional complex. The four textiles were treated with artificial sweat (see Section 2.4. for details) and the resulting solutions were submitted to the Folin–Ciocalteu assay for six consecutive cycles as described in Section 2.5. Plots in Figure 3 reveal that, in correspondence of the first cycle, the highest release was provided by sample T3, while all the other samples displayed a similar behavior. At the second cycle, a comparable release was observed for the three textiles submitted to the mordanting treatment (T3–T5), which was higher than that measured for the not-mordanted sample (T1).

At the third cycle, negligible differences were observed for all the four textiles, which exhibited an identical behavior from cycle fourth on. Based on the initial highest release by T3, this sample was subsequently selected for the biological studies described in Section 3.4.

#### 3.2.2. Determination of Polyphenols by HPLC-UV/Vis

**Figure 4:**
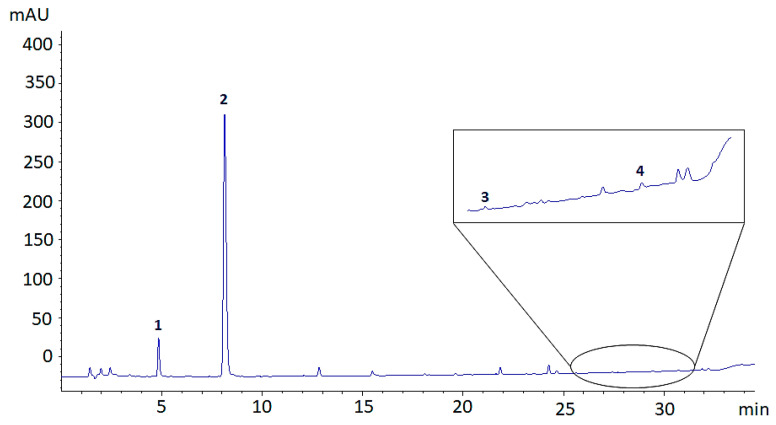
Representative HPLC-UV chromatogram of artificial sweat containing polyphenols released from a wool yarn dyed with the onion skin extract. For peak identification: 1. Protocatechuic acid glucoside, 2. Protocatechuic acid, 3. Quercetin-4′-glucoside, 4. Quercetin. Glycosylation site was tentatively assigned on the basis of the literature data.

Given quantitative results on total polyphenols by means of the Folin-Ciocalteau assay, only samples T1–T3 and T6 were considered fur further analysis by means of HPLC-UV/Vis. Figure 4 shows the representative HPLC chromatogram of a sample of dyed textile after artificial sweat release, recorded at 254 nm. As it is possible to observe, the main peaks on the chromatogram are related to the presence of protocatechuic acid and quercetin derivatives, with the former being the most abundant.

Quantitative data related to the release of polyphenols by dyed textiles in artificial sweat, determined by means of HPLC-UV/Vis, are shown in Table 3. Being that all detected compounds are either protocatechuic acid or quercetin derivatives, they were all quantified either as protocatechuic acid or quercetin equivalents, respectively. Results in Table 3 suggest a higher release of protocatechuic derivatives from the textile, with respect to quercetin-like compounds. This is in accordance with the quali- and quantitative profile of polyphenols in onion ethanolic extract and H_2_O residue from the dyeing process described in previous works [5,8], where protocatechuic acid was found to be the most abundant phenolic compound, though a higher amount of quercetin derivatives was generally detected [5]. This slight difference in the polyphenolic profile is not surprising, as protocatechuic acid (having a pK_a_ of 4.48 [30]) at pH 6.5 is dissociated and, therefore, it displays a higher solubility in the aqueous medium, contrary to quercetin. In contrast to what is expected, also quercetin-4′-glucoside was not released in a great extent, despite its polar nature; this observation may lead to the conclusion that quercetin glycosides might be more retained by the textile with respect to protocatechuic acid and its glycosylated derivatives.

On the basis of all the above, sample T3 was selected for all the following chemical and biological evaluations.

### 3.3. Determination of Alum Cytotoxicity on RAW 264.7 Cells

To evaluate the impact of alum on cell viability of RAW264.7, a macrophage cell line commonly used to determine the potential toxicity of biologically active compounds was here employed. Cells were left untreated or treated by using the same amount of alum used in the dyeing procedure.

**Figure 5:**
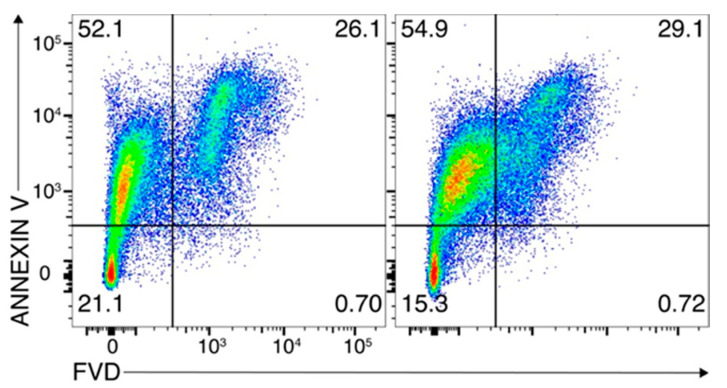
RAW264.7 cells were left untreated (left panel) or treated for 24 h with alum. Cells were stained using the PerCP-Annexin V and FVD 780 and analyzed by flow cytometry. Annexin V/FVD—double negative cells (lower left quadrants) represented live cells, annexin V/FVD—double positive cells (upper left and upper right quadrants) represented apoptotic cells, and annexin V-negative/FVD-positive cells (lower right quadrants) indicated dead cells. Data are representative of two different experiments.

After 24 h of incubation, cell viability and apoptosis were evaluated by means of a cytofluorimetric analysis (Figure 5). The analysis revealed that the percentage of live (lower left quadrants), dead (lower right quadrants), and apoptotic cells (upper right and upper left quadrants, indicating early and late apoptotic cells, respectively) were almost overlapped in the two samples, showing that alum is safe for RAW264.7 cell line at the concentration used in the dyeing method.

### 3.4. Effects of Biofunctional Textile on Intracellular and Extracellular ROS Production

**Figure 6:**
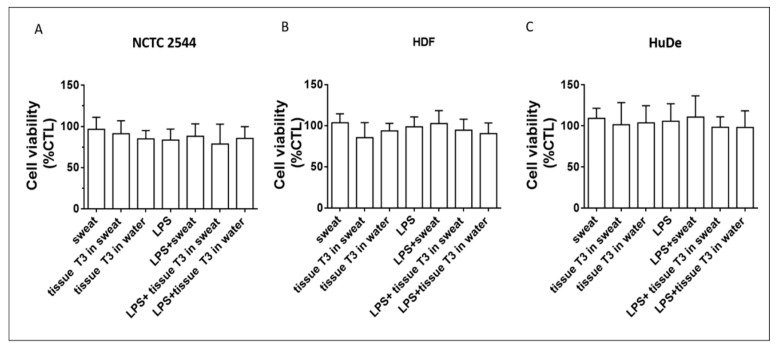
Effects of tissue T3 on cell viability of skin cells. The effects of tissue T3 in artificial sweat and H_2_O on cell viability were assessed after 24 h of incubation using the MTT test in human keratinocytes, NCTC 2544 (**A**), HDF, primary human dermal fibroblasts (**B**), and HuDe, human dermal fibroblasts (**C**). Cells were pre-treated with LPS for 6 h. Cell viability was expressed as % of untreated cells (CTL). Data are expressed as mean ± SD of three independent experiments carried out in triplicate.

**Figure 7:**
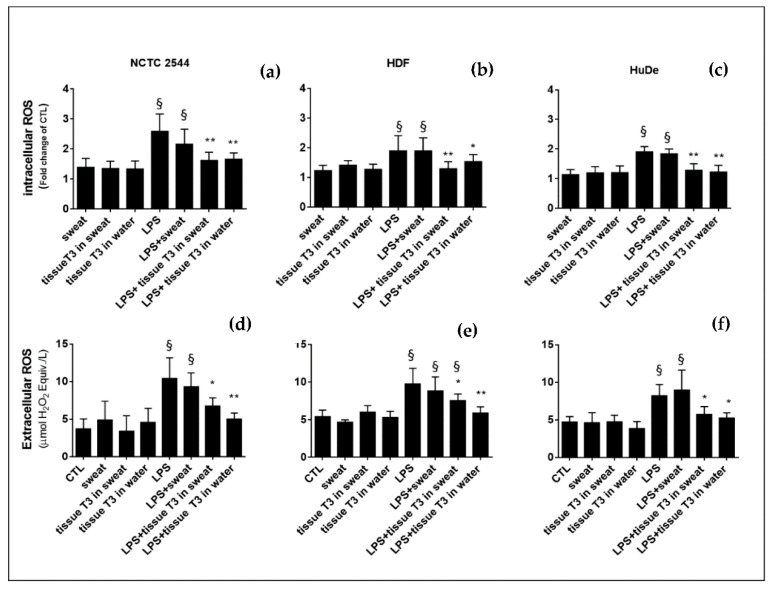
Effect of sample T3 on ROS production induced by LPS in human dermal cells. Intracellular levels of ROS (iROS) were measured by the fluorescent probe DCFH-DA in (**a**) NCTC 2544, (**b**) HDF, and (**c**) HuDe cells pre-treated or not with LPS for 6 h and then treated with sample T3 in artificial sweat or H_2_O for 24 h. Extracellular ROS in the cell medium were measured utilizing the Amplex Red probe: (**d**) NCTC 2544, (**e**) HDF, and (**f**) HuDe cells pre-treated or not with LPS for 6 h and then treated with sample T3 in artificial sweat or H_2_O for 24 h. To validate the assay, quality control tests (QC) were carried out spiking the samples with known concentrations of H_2_O_2_. Data were mean ± SD of three independent experiments run in triplicate. One way ANOVA: CTL vs. treatments ^§^ *p* < 0.05; LPS vs. LPS + treatments * *p* <0.05; ** *p* < 0.01.

Human skin cells were pre-treated with the bacterial wall component LPS to induce an antigenic response and inflammatory gene activation. Then, cells were treated with the T3 phenol-rich extract in artificial sweat or H_2_O for 24 h for the determination of cell viability (Figure 6), together with both intra- and extra-cellular ROS levels (Figure 7). As already anticipated in Section 3.2.1, only extract from sample T3 was considered for the biological evaluations here described. Both extracts from T3 in artificial sweat and in H_2_O did not show any toxic effect after 24 h of treatment in keratinocytes (NCTC 2544) (Figure 6A) and in human dermal fibroblasts (HDF and HuDe) (Figure 6B,C) pre-treated and not for 6 h with LPS.

The extract from T3 in both artificial sweat and in H_2_O showed a significant reducing effect on the level of both intracellular (Figure 7a–c) and extracellular (Figure 7d–f) ROS in human keratinocytes and dermal fibroblasts pretreated with LPS. Therefore, the extracts from the T3 textile showed a good antioxidant capacity in vitro using an LPS-induced cellular stress model in skin cells.

## 4. Conclusions

In the present study, onion skin from the *Dorata di Parma* cultivar was evaluated as a source of valuable phenolic compounds to be potentially exploited in the biofunctional textile industry. Wool yarn was selected as the textile and dyed with onion skin aqueous extracts obtained either in the presence or in the absence of additives (that is, either a zwitterionic surfactant or a mordanting agent). The use of alum as a mordant in the dyeing process was evaluated with three different application methods. The uptake of bioactive compounds by wool yarns and their release into artificial sweat in standard conditions was monitored by means of both UV/Vis spectroscopic and HPLC-UV/Vis analyses. Among the different mordanting techniques, the pre-treatment of wool with alum (sample T3) led to the highest release efficiency of phenols in an artificial sweat solution, as demonstrated by its highest content of protocatechuic acid and quercetin equivalents determined by means of HPLC-UV/Vis analysis.

The cytofluorimetric analysis demonstrated that the employed alum mordant did not have any significant influence on cell viability and apoptosis RAW264.7 cells, thereby indicating that the mordant treatment is safe at the concentration used in the dyeing procedure.

The manufactured material was also evaluated for its in vitro antioxidant capacity. The extract released from the manufactured textile immersed in artificial sweat showed a significant reducing effect on both the intracellular (up to 37%) and extracellular (up to 30%) ROS levels in human keratinocytes and dermal fibroblasts pre-treated with LPS.

In conclusion, this work highlights the biological potential of onion skin waste and its real possible application to textile industry for the production of new-generation sustainable textiles in a circular economy perspective.

## Figures and Tables

**Table 1:** Colorimetric coordinates of textile samples.

Sample	L*	a*	b*
T1	55.44 ± 0.85	13.48 ± 0.46	25.53 ± 0.47
T2	50.24 ± 2.21 ***	15.26 ± 0.05 **	23.39 ± 0.53 *
T3	55.29 ± 0.09 **	13.93 ± 0.14	33.15 ± 0.68 ***
T4	54.14 ± 1.91	12.58 ± 0.04 *	31.54 ± 1.85 ***
T5	66.59 ± 0.17 ***	6.17 ± 0.36 ***	48.70 ± 0.65 ***
T6	48.46 ± 0.98 ***	13.68 ± 0.44	30.7 ± 2.31 ***

Data are expressed as mean ± SD of three independent experiments. One-way ANOVA test analysis was performed evaluating the difference of textiles T1 vs. T2–T6. As a result, the colorimetric index L* of T1 was found to be statistically different from all samples (*** *p* < 0.0001, ** *p* < 0.005) except for T4. Concerning the a* index, T1 was found to be statistically different from samples T2 (** *p* < 0.005), T4 (* *p* < 0.05), and T5 (*** *p* < 0.0001). The b* index concerning T1 was found to be statistically different from all samples.

**Table 2:** Total phenol content of textile extracts as determined by using the Folin–Ciocalteau method. Data are expressed as mgGAE/g textile ± SD.

Sample	mgGAE/g Textile
T1	17.3 ± 0.7
T2	21.8 ± 2.8
T3	21.8 ± 0.3 ***
T4	18.0 ± 1.3 *
T5	11.3 ± 3.1 *
T6	20.7 ± 1.6

Data are expressed as mean ± SD of three independent experiments. A student’s *t*-test analysis was performed evaluating the difference of textiles T2–T6 vs. T1. As a result, T1 was found to be statistically different from T3 (*** *p* < 0.001), T4 (* *p* < 0.05), and T5 (* *p* < 0.05).

**Table 3:** Quantitative data of the main polyphenols released in artificial sweat solution by wool tissues dyed with the onion extract. Data are expressed as µg/mL ± SD of protocatechuic acid and quercetin equivalents.

	T1	T2	T3	T6
Protocatechuic acid equivalents	16.2 ± 1.0	19.1 ± 0.3	17.6 ± 2.2	15.8 ± 0.5
Quercetin equivalents	2.7 ^a^	2.9 ± 0.1	2.8 ± 0.1	2.9 ± 0.1

Data are expressed as mean ± SD of three independent runs. ^a^ SD < 0.05.

## Data Availability

The data presented in this study are available in this manuscript.
